# *Scirpus radicans* (Cyperaceae), a newly-discovered native species in Lithuania: population, habitats and threats

**DOI:** 10.3897/BDJ.9.e65674

**Published:** 2021-04-06

**Authors:** Zigmantas Gudžinskas, Laurynas Taura

**Affiliations:** 1 Nature Research Centre, Institute of Botany, Vilnius, Lithuania Nature Research Centre, Institute of Botany Vilnius Lithuania

**Keywords:** assessment, communities, conservation, IUCN Criteria, population size, *Scirpetum radicantis*, vegetative reproduction

## Abstract

A previously unrecorded native species, *Scirpus radicans* (Cyperaceae), was discovered in the southern part of Lithuania in 2020. Although this species has a large distribution area in other parts of Eurasia, it is currently recognised as threatened in many European countries. Recordings of *S. radicans* in Lithuania had been expected, as these plants do occur or have been reported to occur in neighbouring regions. The aim of this study was to analyse the distribution of *S. radicans* in southern Lithuania, to determine the occupied areas and the size of populations, to study their capacity of vegetative reproduction, their habitats and associated plant communities, as well as to perform an assessment of the species' conservation status within the country. We studied *S. radicans* populations at two sites on the shores of Lake Pabezninkai and of Lake Netečius in the Varėna District (southern Lithuania), from August to October 2020. A total of 993 individuals of *S. radicans* were counted and their stands comprised 0.73 ha. The density of individuals in the studied plots ranged from 0.02 to 0.78 plants/m^2^. Only 0.5% of individuals at Lake Pabezninkai and 20.7% of individuals at Lake Netečius were at the stage of generative reproduction. Individuals at the stage of vegetative reproduction comprised 56.4%, whereas generative individuals amounted to 1.7% of all studied plants. One to seven stolons developed per plant and the mean number of rosettes per stolon was 2.8 ± 1.2. Potentially, a total of ca. 2860 rosettes could be expected from all individuals of the studied plots, but the actual realised rate of vegetative reproduction is unknown. A more detailed study of the reproductive capacities of *S. radicans* would be required for better understanding the causes of the lately observed decrease of this species in most of the countries of Central Europe. Our analysis of the associated plant communities has enabled us to confirm the presence of a *Scirpetus radicantis* Nowiński 1930 association previously not recorded in Lithuania. By assessing *S. radicans* in accordance with the IUCN Criteria, we conclude that this species should be classified as endangered (EN) in Lithuania. Based on this assessment, we propose to include this species on the list of legally protected species of Lithuania. Conservation of shallow lakes with sandy or muddy shores and with significant natural fluctuations of the water level is the main measure for ensuring the survival of *S. radicans*, as well as other rare and endangered species adapted to such a type of habitat.

## Introduction

Documenting the diversity, the distribution and the state of populations of plant species in a territory is the first and most fundamental step for effective conservation of biodiversity at the species, habitat and ecosystem level (Sechrest and Brooks 2002). The structure of the flora in almost any territory is constantly changing because of natural factors (Essl et al. 2015, Hilmers et al. 2018); however, recent human activities variously have been leading to habitat destruction (Young et al. 2005, Chase et al. 2020), biological invasions (Ehrenfeld 2010, Simberloff et al. 2013) and climate change (Bolte et al. 2010, Sletvold et al. 2013, Gudžinskas et al. 2016, Hoffmann et al. 2019, Salick et al. 2019).

Currently, perhaps a third of all vascular plant species in certain parts of their habitats or in their entire range are considered as rare, endangered or at risk of extinction (Sechrest and Brooks 2002, Corlett 2016). Thus, it is important that rare and protected plant species are investigated to evaluate the state of their populations, reveal threats and identify conservation measures (Kricsfalusy and Trevisan 2014, Gudžinskas et al. 2016). Attempts to assess the functional importance of the effects of rare species on the integrity of ecological processes in ecosystems have been made (Leitão et al. 2016). The results of such studies have indicated a critical role of rare species in maintaining ecosystems under the ongoing rapid environmental transitions.

The diversity of vascular plant species in Lithuania is well studied (Gudžinskas 1999), while a small number of extremely rare native plant species has remained unnoticed in particular habitats despite thorough floristic studies. Therefore, new native species sometimes are being discovered (Gudžinskas and Sinkevičienė 2002, Sinkevičienė 2011, Sinkevičienė 2013) or re-discovered after a long time after being considered extinct (Sinkevičienė 2016). Our studies in particular unique habitats in 2020 have led to the discovery of *Scirpus radicans* Schkuhr (Cyperaceae) in south Lithuania, a species that has been anticipated to occur in our country, but previously had never been recorded (Snarskis 1954, Gudžinskas 1999).

*Scirpus radicans* is native to the temperate regions of Eurasia with an extensive distribution range (Hulten and Fries 1986). In Europe, this species occurs from France to the eastern part of European Russia and from the southern regions of Norway, Sweden and Finland to Italy and the Balkan peninsula (Pignotti 2003, Jiménez-Mejías and Luceño 2011, Dítě and Eliáš 2013). In Asia, its range stretches from the Urals to eastern Siberia, the Korean peninsula and Japan; the range in Asia is highly disjunctive, though and consists of many quite small, isolated populations (Hulten and Fries 1986, Songyun and Tucker 2010, Chang et al. 2014).

*Scirpus radicans* occupies shallow inland aquatic habitats along riverbanks, shores of lakes and oxbows with strongly fluctuating water level and is regarded as a pioneer species (Zahlheimer 1979, Burkart 2001, Dítě and Eliáš 2013, Więcław et al. 2019). The plants grow in stagnant and periodic flow-through wetlands on unconsolidated clay sediments and occasionally inhabit wet anthropogenic habitats, such as fishponds (Spałek 2005, Hroudová et al. 2011, Dítě and Eliáš 2013). However, habitats favourable for *S. radicans*, frequently are transient because they are quickly occupied by dense common reed stands or, in the absence of natural disturbances, by tall nitrophilous herbs, willows and various shrubs (Hroudová et al. 2011).

Under favourable conditions, *S. radicans* forms communities of the *Scirpetum radicantis* Nowiński 1930 association (class *Phragmito-Magnocaricetea* Klika in Klika et Novák 1941, alliance *Eleocharito palustris-Sagittarion sagittifoliae* Passarge 1964). Communities of this association have been recorded in Central Europe, Ukraine, European Russia and southern Scandinavia (Hroudová et al. 2011, Landucci et al. 2020).

Information about the biological characteristics of *S. radicans* is quite scarce. It is known that this species develops after lowering of the water level when the bottom becomes exposed, so that seeds stored in the soil seed bank can germinate (Hroudová et al. 2011). It is supposed that seeds in the soil seed bank retain their viability for several decades, though generative reproduction is quite limited (Hroudová et al. 2011, Dítě and Eliáš 2013). We were not able to locate any published results of studies on the intensity and success of the vegetative reproduction of *S. radicans*.

Although *S. radicans* according to the IUCN Criteria has been evaluated as a data deficient species at the European Union scale (Bilz et al. 2011), in many European countries, it is included on lists of protected species. In the Czech Republic (Grulich 2012), Hungary (Király 2007) and in the Wielkopolska Region of Poland (Jackowiak et al. 2007), it has been considered as a vulnerable species, whereas in Slovakia (Dítě and Eliáš 2013) and Finland (Hakalisto et al. 2000, Rassi et al. 2010), it is treated as endangered. In Estonia (Mäemets 2016) and Poland (Kaźmierczakowa 2016), *S. radicans* has been found to be a near-threatened species, whereas in Latvia, it is classified as a rare species and has been included in the list of protected plant species (Eglīte 2003). In the Kaliningrad Region of Russia (Dedkov et al. 2010), *S. radicans* has been determined to be an extinct species. However, there is a lack of knowledge about important ecological and biological properties of this species, particularly about its reproductive behaviour. This information is of particular importance for understanding the dynamics of populations and selection of adequate conservation measures.

The aim of this study was to estimate the state of the newly-discovered native plant species *S. radicans* in Lithuania. In this study, we aimed to: (i) investigate the distribution of *S. radicans* in south Lithuania; (ii) evaluate occupied areas and the size of populations at the individual sites; (iii) estimate the reproductive capacity of this species in different stands; (iv) analyse the occupied habitats and plant communities in Lithuania; (v) assess the species conservation status in Lithuania according to the IUCN Criteria.

## Material and methods

### Study area

The study area in southern Lithuania is located in the Varėna District at the southern edge of the Dzūkai upland, which is a part of the Baltic Uplands. The relief of the area is dominated by moraine hills combined with limnoglacial formations (Basalykas 2014). The standard mean annual temperature in the study area is 6.8°C. The coldest month in the area is January with a standard mean temperature of –3.7°C, whereas July is the warmest month with a mean temperature of 17.9°C. The standard mean annual sum of precipitation is 701 mm with the largest amount of precipitation occurring during the summer months (242 mm) and the lowest amount of precipitation falls in winter (145 mm). The mean standard duration of annual sunshine is 1691 hours in the region (Galvonaitė et al. 2013).

Lake Pabezninkai is of glacial origin and occupies an area of 61.4 ha. The Lake is slightly elongated, 1.0 km long and 0.7 km wide. The altitude of the Lake surface is 135 m above sea level. It is a shallow Lake with a mean depth of 1.9 m and a maximum depth of 3.0 m. It is currently classified as a eutrophic lake. The banks of the Lake are low, with gently sloping sandy shores. The Lake is characterised by significant periodical fluctuations of the water level. At the peak of water level drop by 1.5–1.6 m, the area of the lake shrinks to 38.2 ha, exposing sandy or muddy flats 30–100 m wide. Lake Netečius is situated 3.5 km northeast of Lake Pabezninkai, while, in contrast to the latter, being of thermokarst origin. It occupies an area of 90.9 ha and is bell-shaped. The Lake is 1.4 km long and 1.1 km wide. The altitude of the Lake surface is 135.7 m above sea level. The Lake is quite shallow, with a mean depth of 1.9 m and a maximum depth of 4.5 m. Currently this Lake is classified as mesotrophic.

### General provisions

Historical information on *S. radicans* in Lithuania, published in literature, was screened and assessed; we analysed herbarium specimens deposited at the herbaria of the Institute of Botany of the Nature Research Centre (BILAS) and at Vilnius University (WI). Voucher specimens of *S. radicans* collected during this research were deposited at the Herbarium of the Institute of Botany of the Nature Research Centre, Vilnius (BILAS). The nomenclature of vascular plant taxa follows the Euro+Med PlantBase (2021). The nomenclature of syntaxa follows Hroudová et al. (2011) and Landucci et al. (2020).

A distribution map of *S. radicans* in Lithuania was compiled by applying a system of grid cells, which were arranged according to geographical coordinates with sides of 6' of latitude and 10' of longitude. All localities recorded in the same grid cell were marked by one symbol on the map. Assessment of the threat for *S. radicans* populations in Lithuania were performed following the IUCN Guidelines and Criteria (IUCN 2012; IUCN 2017) and based on the results of this study.

### Field studies

Field studies on the distribution, communities and habitats, the size and structure of populations and reproductive behaviour of *S. radicans* were performed from August to October 2020. The area of plots with *S. radicans* was calculated using online software provided from the Spatial Information Portal of Lithuania (www.geoportal.lt), according to geographical coordinates, established at peripheric points of the plot perimeter. Phytosociological relevés of plant communities with *S. radicans* were performed by applying the Braun-Blanquet (1964) approach and we recorded the cover of vegetation layers as well as the percentage of bare substrate. The area of relevés was 100 m^2^. The number of *S. radicans* individuals in plots was counted systematically while surveying the entire area. Individuals were divided into three groups. Generative individuals had at least one inflorescence and one to several stolons. Individuals with at least one stolon, but without inflorescence, were ascribed to the group of vegetatively reproducing individuals, whereas plants without stolons were considered as young vegetative individuals.

The number of stolons were counted for each recorded generative and vegetatively reproducing individual in all plots, except the plot on the south-western bank. In that plot, we studied 50 vegetatively reproducing individuals. The length of the longest stolon was determined with measuring tape at a precision of 1 cm and the number of developed rosettes on the stolon was counted, regardless of whether they were rooted or not.

### Statistical analyses

The results of the descriptive statistics included mean values and standard deviations (mean ± SD), as well as minimum and maximum values. As the number of studied *S. radicans* individuals in the sampling plots was different, the non-parametric Kruskal-Wallis H-test and the Mann-Whitney U post-hoc pairwise comparisons were applied. Pooled data of the number of stolons, their length and number of developed rosettes per stolon, according to the Shapiro-Wilk test, were distributed non-normally, therefore relationships between these characters were tested by employing Spearman’s rank-order correlation (r_s_). The density of individuals per square metre was calculated by dividing the total number of individuals by the occupied area. All calculations were performed using PAST 3.20 (Hammer et al. 2001).

## Results

### Distribution and size of populations

Any previously-available information on the occurrence of *S. radicans* in Lithuania has been controversial for a long time. Some authors treated this species as not occurring in the country, though expected to be present (Snarskis 1954, Gudžinskas 1999), whereas others included it in the list of species, based on older records (Lekavičius 1963, Tabaka et al. 2003). Lekavičius (1963) included this species as a member of the flora of Lithuania, based on a report by Missuna (1896) and stated that *S. radicans* was recorded in the environs of Svylė village, in Ignalina District, east Lithuania with a note that the occurrence was not confirmed by herbarium specimens. However, analysis of the original publication by Missuna (1896) revealed that the location was misinterpreted by Lekavičius (1963). In the introductory section of his article, Missuna (1896) described the study area as being in the environs of Swiłły farmstead, which was situated ca. 12 km from the Głębokie Village (Glubokoye or Hlybokaye), on the road to Lepel. Thus, Swiłły farmstead (also mentioned as Świłły in the text), where Missuna (1896) had performed his studies on the local flora, was located in the former Dysna County (*powiat Dziśnieński*), whereas Svylė Village, situated in Ignalina District at the end of the 19th century, was in the former Švenčionys County (*powiat Święcianski*). Thus, the locality of *S. radicans*, as reported by Missuna (1896), is located in the current Glubokoye District, in the Vitebsk Region of Belarus. Therefore, *S. radicans* has not been recorded in the current territory of Lithuania and all reports of its occurrence were based on misinterpreted information.

**Figure 1.**
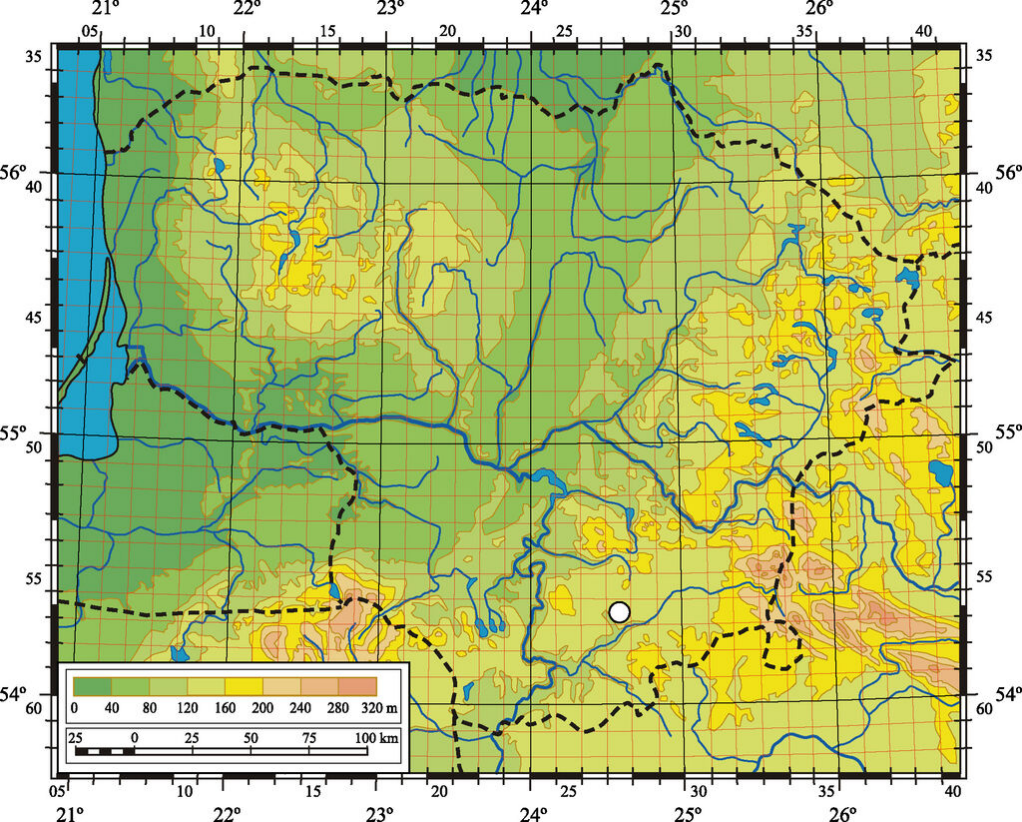
Location of the studied *Scirpus radicans* populations in Lithuania (white dot).

The first locality of *S. radicans* in Lithuania was found in the Varėna District, 2 km southeast of Sarapiniškės Village, in the environs of Aukštakalnis settlement (Fig. 1), on the eastern shore of Lake Pabezninkai (28 August 2020; 54.35265°N, 24.27300°E). A small group of individuals occurred on the wet sandy shore of the lake. The second locality of *S. radicans* was observed ca. 4 km eastwards from the first locality, in the Varėna District, in the environs of Puodžiai Village, on the shore of Lake Netečius. A group of plants was found at the south-eastern shore of the Lake, in the transitional zone between a stand of *Alnus glutinosa* and a belt of *Phragmites australis* (10 October 2020; 54.35647°N, 24.63457°E). Solitary *S. radicans* individuals were also recorded on the south-eastern shore of Lake Netečius, in wet sand with sparse vegetation (10 October 2020; 54.35953°N, 24.62372°E).

**Figure 2.**
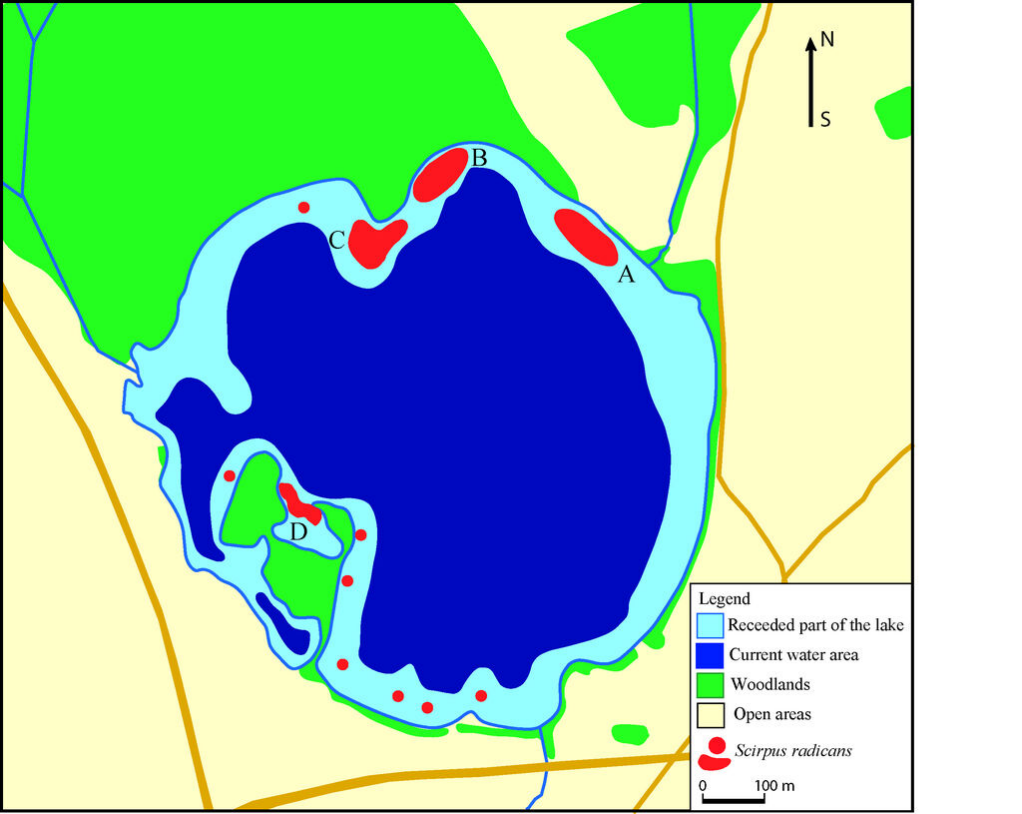
Distribution of *Scirpus radicans* on the shores of Lake Pabezninkai in 2020. Letters (A, B, C and D) indicate study plots.

Thorough surveys of the shores of Lake Pabezninkai comprising ca. 4.2 km revealed four plots with relatively compact stands of *S. radicans*, as well as eight separate localities where solitary – or occasionally 2 or 3 – plants were found (Fig. 2). The total currently-known area, occupied by *S. radicans* stands in Lithuania, covers 0.73 ha. The density of *S. radicans* individuals was low in the north-eastern (plot A) and northern (B) plots (0.03 and 0.02 individuals/m^2^, respectively). The density of individuals was somewhat higher at the north-western (C) plot where the mean density was 0.11 individuals/m^2^, whereas the highest density was recorded at the south-western (D) plot (0.78 individuals/m^2^). It should be noted that young individuals of *S. radicans* without stolons in plots A–C prevailed, whereas most of plants recorded in plot D had stolons (Table 1). On the south-eastern shore of Lake Netečius, *S. radicans* occupied an area of 120 m^2^ and 58 individuals were recorded; thus, its mean density was 0.48 individuals/m^2^. A total of 993 individuals of *S. radicans* were counted on the shores of Lakes Pabezninkai and Netečius.

It is important to note that only five individuals with inflorescences were found on the shores of Lake Pabezninkai and 12 individuals at Lake Netečius. Thus, only 0.5% of the individuals (n = 935) at Lake Pabezninkai and 20.7% of individuals (n = 58) at Lake Netečius were at the stage of generative reproduction. Furthermore, young individuals without stolons comprised 44.2% of all individuals recorded at Lake Pabezninkai and they were prevailing in three plots. Only in plot D did individuals with stolons prevail (Table 1). Young individuals without stolons comprised a quite small part of all plants (8.6%) on the shore of Lake Netečius. Analysis of all studied plants pooled showed that individuals at the generative stage comprised 1.7%, while, in the stage of vegetative reproduction 56.4% and 41.9% of individuals were young, without stolons.

During September and October of 2020, we also screened the shores of Lakes Lavysas and Glėbas, which are characterised by significant fluctuations of the water level as in Lake Pabezninkai; however, we could not find *S. radicans*. The species was not found on the shores of other surveyed lakes of southern Lithuania either (Lakes Glūkas, Juodikis, Kastinis and Pelekas).

### Vegetative reproduction

The vegetative reproduction of *S. radicans* on the shores of Lake Pabezninkai was studied in four plots and we analysed a total of 121 individual plants. Individual plants produced one to six stolons and the mean number of stolons was 2.3 ± 1.4 per plant. Analysis of the number of stolons revealed significant differences between the study plots (Kruskal-Wallis H = 13.22; p = 0.002). Pairwise comparison revealed that, in plot C and D, *S. radicans* produced significantly more stolons than in plot A (Mann-Whitney U = 185.5, p = 0.002 and U = 391.0, p = 0.001, respectively). The largest mean number of stolons per plant was recorded in plot C, whereas the lowest number was in plot A (Table 2).

Mean stolon length varied within a broad range. The shortest recorded stolon was 18 cm, whereas the longest stolon was 141 cm long (Table 2). The analysis revealed no significant differences of the stolon length between all study plots (Kruskal-Wallis H = 2.61; p = 0.455) nor between individual plots in applying Mann-Whitney pairwise comparisons. The mean length of stolons of all studied individuals was 92.7 ± 24.4 cm.

The number of developed rosettes on stolons of the studied individual in all plots ranged from one to seven. The mean number of rosettes per stolon was 2.8 ± 1.2 and we found no significant differences between study plots (Kruskal-Wallis H = 2.55; p = 0.434). Pairwise comparison also revealed no differences in the number of rosettes per stolon between the studied plots (Table 2). Thus, every individual in the vegetative reproduction stage produces 5.52 ± 1.68 rosettes. Each rosette under favourable conditions can take root and, when detached from the parent plant, becomes an independent individual. Considering the total number of recorded individuals at the vegetative reproduction stage (Table 1) and mean number of developed rosettes, ca. 2860 new individuals of vegetative origin might have been produced at Lake Pabezninkai in 2020.

When analysing the pooled data (n = 121) of vegetative reproduction of *S. radicans*, we found a strong reliable correlation between the length of stolons and the number of developed rosettes (r_s_ = 0.78; p < 0.001). However, weak, but reliable, correlations were found between the number of stolons per individual and the length of the longest stolon (r_s_ = 0.42; p < 0.001), as well as between the number of rosettes and the number of stolons per individual (r_s_ = 0.43; p < 0.001).

### Habitats and communities

**Figure 3.**
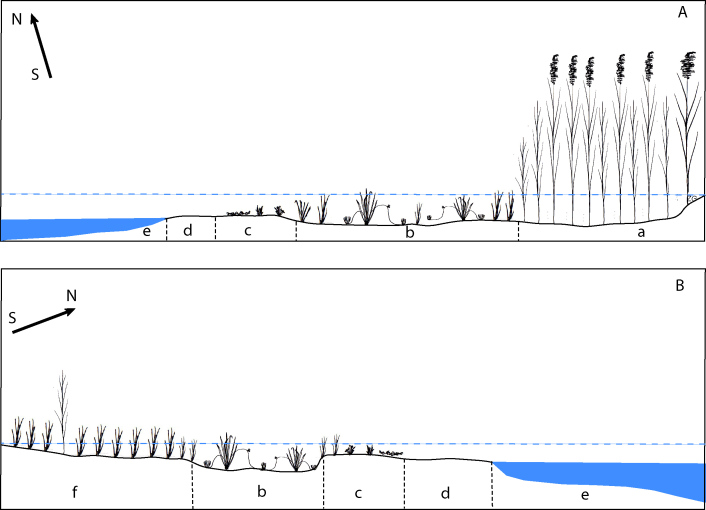
Schemes of habitats occupied by *Scirpus radicans* and its communities on the north-eastern (A) and south-western (B) shores of Lake Pabezninkai. Blue dotted lines indicate usual water level in the lake. The main belts of plant communities and habitats are indicated by lowercase letters: *Phragmites australis* bed (a), communities formed by *Scirpus radicans* (b), communities of therophytes (c), wet bare sand areas (d), lake (e) and *Carex acuta* community (f).

All recorded stands of *S. radicans* and separately growing individuals on the shores of Lake Pabezninkai occupied areas exposed from water because of the drastic decrease (ca. by 1.5 m) of the water level in the Lake. Plants occupied areas of wet silt or sand occasionally with admixture of medium-sized pebbles. Analysis of the sites of *S. radicans* stands revealed that all areas during the period of high water level were inundated and gradually became exposed since the start of the drastic water level decrease in 2018 (Fig. 3). The habitat on the south-eastern shore of Lake Netečius was not affected by significant fluctuations of the water level, as plants occupied areas slightly above the eroded bank of the Lake, along the edge of an *Alnus glutinosa* stand.

Our analysis of the composition and structure of the plant communities in which *S. radicans* occurs on the shores of Lake Pabezninkai (Table 3) represent the *Scirpetum radicantis* Nowiński 1930 association (class *Phragmito-Magnocaricetea* Klika in Klika et Novák 1941, alliance *Eleocharito palustris-Sagittarion sagittifoliae* Passarge 1964). Communities of the *Scirpetum radicantis* association were formed between *Phragmites australis* or *Carex acuta* stands and plant communities dominated by therophytes and swards of dwarf perennial plants developed on wet sand (e.g. *Elatine hydropiper*, *Eleocharis ovata*, *Juncus bufonius*, *J. bulbosus*, *J. articulatus*, *Gnaphalium uliginosum*, *Ranunculus reptans* and *Rorippa palustris*). Communities of the *Scirpetum radicantis* association occupied shallow depressions with wet mud (plot A) or wet sand (plots B, C and D) without standing water (Fig. 3).

The cover of *Scirpus radicans* in the analysed communities was quite uniform, ca. 20%, except for plot D, where its cover was 35% (Table 3). Another species of the association, *Alisma plantago-aquatica*, was present in all relevé plots, but its cover was low in areas with wet sand (plots C and D). The usual characteristic species of the *Eleocharito palustris*-*Sagittarion sagittifoliae* alliance (*Eleocharis ovata*, *E. palustris* and *Juncus articulatus*) were constant and their abundance quite low. Most of the characteristic species of the *Phragmito*-*Magnocaricetea* class were also constant, but were represented by solitary individuals in the relevé plots (Table 3). In the group of accompanying species, the most constant were plants characteristic of open wet habitats (*Agrostis stolonifera*, *Alopecurus geniculatus*, *Bidens tripartita* and *Juncus bulbosus*). The presence of quite many species of therophytes in the analysed communities supports the presumption that the lake shores, accommodating the *Scirpetum radicantis* association, have been exposed for a rather short time, possibly for two or, maximum, three (plot D) years.

### Assessment of conservation status by applying the IUCN Criteria

*Scirpus radicans* currently is registered in two localities in Lithuania. The area of occurrence of this species in Lithuania comprises 4.6 km^2^, whereas the total area of occupancy in both localities comprises just 0.0073 km^2^ (0.73 ha). Furthermore, considering the specific occupied habitats and the nature of Lake Pabezninkai with substantial changes in the water level, significant decreases in the area of occupancy and extreme fluctuations of mature individuals are projected. Thus, according to the criterion of the geographic range (IUCN 2012), *S. radicans* should be considered as a critically endangered (CR) species.

This study has revealed that the population of *S. radicans* in Lithuania is small (the total number of recorded individuals was 993 and 577 of these were mature plants) and a reduction of the population is suspected in the future because of the projected decline of the area of occupancy. The main suspected cause of the decline of the area of occupancy is a change in the water level of Lake Pabezninkai. Two scenarios of possible habitat changes are to be expected: if the water level of the Lake suddenly rises again, the area of suitable habitats would be drastically reduced to 200–300 m^2^ and, therefore, the population would shrink. According to the second scenario, if the water level in the Lake remains the same or falls further, unfavourable successive changes in habitats will begin, mainly because of expansion of *Phragmites australis* stands or shrubby willows. Periodical significant water level changes in Lake Pabezninkai would be the most favourable for the stability of the population of this species, though fluctuations in the number of individuals are still expected. Thus, considering the criterion of *S. radicans* population size and its suspected changes in Lithuania, this species should be assessed as an endangered (EN) species.

Summarising the results of this study on the recently discovered population of *S. radicans* in Lithuania and considering existing as well as inferred threats in the country and in several regions of Central Europe, we conclude that its assessment as critically endangered could be premature. Further studies on the dynamics of the population and surveys of habitat changes are required. Therefore, following the guidelines for applying the IUCN Criteria (IUCN 2012), we preliminarily assess *S. radicans* as an endangered species in Lithuania (**EN A3c; B2a; C1+2b**).

## Discussion

### Distribution and size of population

Although the distribution range of *S. radicans* generally includes Lithuania (Hulten and Fries 1986) and one has expected to find this plant also in this particular country (Snarskis 1954, Gudžinskas 1999), only the recent discovery of its population confirms its occurrence – though the species appears to be rare in the country. It is known that certain species can be rare because they have small geographic ranges, narrow habitat tolerances, small populations or any combination thereof (Harnik et al. 2012). Thus, we suppose that the rarity of *S. radicans* in Lithuania is caused by the combination of quite a narrow habitat tolerance and, thus, only small populations. Despite the search for *S. radicans* on the shores of several other lakes with similar habitat conditions (significant water level fluctuations, exposed sandy and muddy shores) in southern Lithuania, this species was recorded at two nearby lakes only. Populations may exist in a few still unknown localities in Lithuania, particularly in the southern and eastern regions of the country, but a significantly wider distribution is not to be expected. This presumption is supported by the known distribution of *S. radicans* in neighbouring regions of Poland (Zając and Zając 2001), Belarus and Latvia (Eglīte 2003, Priedītis 2014). Furthermore, the plant is treated as extinct in the neighbouring Kaliningrad Region of Russia (Dedkov et al. 2010).

Information on the size or density of *S. radicans* populations from different parts of the range is scarce, except for scattered data on the occupied areas by its stands in several Central European countries. Spałek (2005) has reported three localities of *S. radicans* in south-western Poland, occupying 0.2 ha, 0.25 and 0.5 ha. The size of a stand of *S. radicans* in Slovakia has been reported to be even smaller, occupying 0.015 ha (Dítě and Eliáš 2013). Thus, the stands of this species on the shores of Lake Pabezninkai and Lake Netečius (Table 1) appear to be comparable with those stands in Poland and Slovakia. Data on the number of individuals and their density in most localities of Central Europe are absent. The results of our study suggest that the density of *S. radicans* in different plots depends on the age of the stand. In those studied plots with recently-receded water (plots A, B and C on the shores of Lake Pabezninkai), the density of individuals ranged from 0.03 individuals/m^2^ to 0.11 individuals/m^2^, whereas, in plots exposed from water for a longer time (plot D), the density was 0.78 individuals/m^2^ (Table 1, Fig. 2). Stands of *S. radicans* develop from the seed bank on recently-exposed shores and then become denser because of vegetative reproduction by stolons (Hroudová et al. 2011, Kaplan et al. 2018). However, the density of *S. radicans* significantly decreases or the plants disappear completely after the water level rises (Hroudová et al. 2011).

### Vegetative reproduction

The survival of rare and endangered plant species and the stability of their populations depend upon the success of their reproductive features in the particular environmental context (Lander et al. 2019). Therefore, selection of conservation measures for species and management of habitats should be based on knowledge about their reproductive and population dynamics (Aronne 2017). The results of our study showed that the generative reproduction of *S. radicans* in Lithuania was extremely poor in 2020. Only 1.7% of all recorded individuals were in the generative stage, whereas most of the individuals (58.1%) were at the stage of vegetative reproduction. Thus, vegetative reproduction plays the main role for increasing the number of individuals at this stage of population development. We found that the mean number of developed stolons was significantly larger in plots that emerged from water at an earlier time, thus, supposedly were composed of more mature individuals. The mean number of developed stolons was the lowest for individuals in the plot that had emerged from the water in the season of study. However, no significant differences were found between study plots with respect to the mean length of stolons and mean number of developed rosettes.

In our investigation, an individual of *S. radicans* at the stage of vegetative reproduction was shown to produce 5.52 ± 1.68 rosettes. Thus, the potential rate of vegetative reproduction can be stated as high, though the rate of realised vegetative reproduction was not evaluated in this study and we were not able to find published information on the matter. Some of the developed rosettes that touch the ground early in the season developed roots, detach from the parental plant and become independent individuals. However, the fate of rosettes that remain above ground level for a longer period of time is unknown. Nevertheless, vegetative reproduction plays a significant role in the fast occupation of areas exposed from water (Hroudová et al. 2011, Kaplan et al. 2018). We suppose that limited sexual reproduction could be amongst the reasons of the restricted species distribution in Lithuania. More detailed studies on the vegetative reproduction of *S. radicans* should be able to reveal its effect on the development and survival of populations under changing habitat conditions caused by water level fluctuations.

### Habitats and communities

In the European part of the range, *S. radicans* usually occurs in fertile, muddy, clayey or sandy soils on riverbanks, along shores of standing waters, in fishponds and other habitats with periodically inundated disturbed soils (Zahlheimer 1979, Hroudová et al. 2011, Dítě and Eliáš 2013, Kaplan et al. 2018). In the Asiatic part of its range, this species occupies similar habitats, though information on habitat preferences is scarce (Park and Kim 2020). The reported habitats of *S. radicans* on the shores of Lake Pabezninkai in Lithuania were typical for this species. It occupied temporarily exposed wet sandy and muddy shores of the Lake. However, a somewhat different kind of habitat was occupied on the shore of Lake Netečius, where it was found in a transitional zone between an *Alnus glutinosa* stand and the shoreline belt of *Phragmites australis*. The latter habitat was without any evident recent natural or artificial disturbances, though it could be affected occasionally by a rising water level in spring or autumn. Although the stand of *Scirpus radicans* was quite small and occupied the entire open area (120 m^2^), this fact suggests that, under certain conditions, this species can survive in undisturbed or almost undisturbed sites. However, most researchers claim that optimum conditions for this species are in areas with recurring disturbances (Zahlheimer 1979, Hroudová et al. 2011, Dítě and Eliáš 2013, Kaplan et al. 2018).

The *Scirpetum radicantis* Nowiński 1930 association has been identified in Central and Eastern Europe, while only few scattered localities have been reported from Northern Europe (Hroudová et al. 2011). Thus, the occurrence of this association in Lithuania was likely. Although *S. radicans* occurs in Latvia and Estonia (Priedītis 2014, Mäemets 2016), information on phytosociological relationships of communities with this species in these countries is missing. Communities of this association in Poland (Spałek 2005, Spałek 2017) and Slovakia (Dítě and Eliáš 2013) frequently were impoverished and composed of four to nine species, including the dominant *S. radicans*, whereas, in the Czech Republic (Hroudová et al. 2011), the community consisted of a few more species (7–15 species). The *Scirpetum radicantis* association in Lithuania was significantly richer in the total number of plant species (from 17 to 29), including four to six species characteristic of the *Eleocharito palustris*-*Sagittarion sagittifoliae* alliance and four to eight species characteristic of the *Phragmito-Magnocaricetea* class (Table 3). We suppose that species richness of the *Scirpetum radicantis* association in Lithuania may depend on the natural condition of habitats, whereas impoverished communities in Poland and Slovakia have been reported to be located in aquatic or secondary habitats (Spałek 2005, Spałek 2017, Dítě and Eliáš 2013). The natural condition of the habitats on the shores of Lake Pabezninkai is indirectly indicated by the presence of rare plant species, such as *Elatine hydropiper*, *Eleocharis ovata*, *Juncus bulbosus* and *Ranunculus reptans*.

### Assessment of conservation status by applying the IUCN Criteria

Our assessment of the here reported *Scirpus radicans* population in Lithuania on the basis of the IUCN (2012) Criteria leads us to consider that this species should be classified as endangered (EN). In most countries of Central and North Europe, *S. radicans* has been assessed as vulnerable (Jackowiak et al. 2007, Király 2007, Grulich 2012) or endangered (Hakalisto et al. 2000, Rassi et al. 2010, Dítě and Eliáš 2013). In Estonia, this species has been assessed as near threatened (Mäemets 2016). Therefore, our assessment not only reflects the state of the population of this species in Lithuania, but also confirms trends in the decline of this species in the European part of its range.

*Scirpus radicans* occurs in habitats with quite strict ecological requirements; therefore, any decrease in available habitats and changes in their quality are amongst the most serious threats for its occurrence in most parts of Central Europe (Király 2007, Grulich 2012, Dítě and Eliáš 2013). Probably the same threats exist in other parts of its range. Open sandy and muddy habitats along natural riverbanks, though not rare, are usually very dynamic and temporal. Nitrogen pollution has remarkably increased in most aquatic environments over the past decades (Galloway et al. 2004); this, along with other biogenic substances, has started to modify coastal habitats which are increasingly being occupied by invasive or other fast-spreading plant species. Shallow inland water and shoreline habitats, similar to coastal bays and lagoons, are particularly vulnerable to rapid changes (McGlathery et al. 2007, Brauns et al. 2011). Therefore, selection of conservation measures for *S. radicans*, as well as for other threatened species with similar biological features and ecological requirements, becomes even more complicated.

Shallow lakes with significant natural fluctuations of the water level are rare; even rarer are lakes whose sandy or muddy shores are free or, to some extent, free from tall helophytes. Thus, conservation of habitats hosting plant species and their communities adapted to periodically inundated conditions – as those along the shores of Lake Pabezninkai – is important to ensure the survival of *S. radicans*, as well as other rare and endangered species (*Elatine hydropiper*, *Eleocharis ovata*, *Juncus bulbosus* and *Ranunculus reptans*).

## Conclusions

*Scirpus radicans* has recently been discovered in Lithuania. The plant is clearly distinguishable and easily identified. It appears to be a rare native species in Lithuania. In a thorough inventory at the recorded sites, we found the total population of this species to consist of less than 1000 individuals. Although *S. radicans* reproduces vigorously by stolons, generative reproduction is extremely limited. Therefore, further studies are needed on the hitherto insufficiently investigated vegetative and generative reproduction of this species in Lithuania, as well as in other regions of Europe. This knowledge would be important for better understanding the causes of decline of its population size and frequency of occurrence in most Central European countries.

The discovery of *S. radicans* in Lithuania allows us to confirm the occurrence of the *Scirpetum radicantis* association at the sites where this plant occurs. An important feature of the recorded communities is the presence of many characteristic species of the *Eleocharito palustris*-*Sagittarion sagittifoliae* alliance and the *Phragmito*-*Magnocaricetea* class, as well as a high total species richness.

Based on the results of this assessment, we propose to include *Scirpus radicans* in the list of legally protected species of Lithuania. This would provide a legal basis for applying conservation measures regarding the habitats, communities and the entire ecosystem of Lake Pabezninkai, as well as the sites of its occurrence on the shores of Lake Netečius. We also suggest considering the establishment of a nature protection area with inclusion of Lake Pabezninkai and the surrounding areas, based on the exceptional hydrological regime of the Lake, as well as on the particular regional occurrence of many rare and protected plant species.

## Figures and Tables

**Table 1.** The number of *Scirpus radicans* individuals by maturity stages and their densities in the studied plots in 2020. Locations of the study plots on the shores of Lake Pabezninkai are shown in Fig. 2.

**Sites and study plots**	**Lake Pabezninkai**					**Lake Netečius**	**Total**
	**A**	**B**	**C**	**D**	**Dispersed**		
Generative individuals	0	2	0	3	0	12	17
Vegetatively reproducing individuals	27	18	26	442	6	41	560
Young vegetative individuals	38	46	70	249	10	5	418
Total number of individuals	65	64	96	694	16	58	993
Occupied area (m^2^)	2490	2960	880	890	–	120	7340
Density (individuals/m^2^)	0.03	0.02	0.11	0.78	–	0.48	0.14

**Table 2.** Evaluation of vegetative reproduction of *Scirpus radicans* around Lake Pabezninkai in 2020. Different lowercase letters denote statistically significant differences between the mean number of stolons in applying the Mann-Whitney post-hoc test.

**Study plots**	**Number of studied plants (n)**	**Number of stolons**		**Stolon length (cm)**		**Number of rosettes per stolon**		
		**Mean ± SD**	**Min–Max**	**Mean ± SD**	**Min–Max**	**Mean ± SD**		**Min–Max**
A	27	1.7 ± 1.3 a	1–6	88.1 ± 29.2	19–135	2.6 ± 1.5		1–7
B	18	2.2 ± 1.3 ab	1–5	86.4 ± 30.8	18–118	2.8 ± 1.1		1–4
C	26	3.0 ± 1.6 b	1–6	97.8 ± 22.6	37–129	3.0±1.1		1–5
D	50	2.4 ± 1.1 b	1–5	94.7 ± 19.0	53–141	2.8 ± 1.1		1–5
**Pooled**	**121**	**2.3 ± 1.4**	**1–6**	**92.7 ± 24.4**	**18–141**	**2.8 ± 1.2**		**1–7**

**Table 3.** Composition of communities of the *Scirpetum radicantis* Nowiński 1930 association on the shores of Lake Pabezninkai (South Lithuania) in 2020. Relevé plots marked with letters correspond to the plots of *Scirpus radicans* presented in Fig. 2.

Relevé plots	A	B	C	D
Cover of the herb layer (%)	70	60	40	70
Bare soil (%)	30	40	60	30
**Ch. & D. *Scirpetum radicantis***				
*Scirpus radicans*	2	2	2	3
*Alisma plantago-aquatica*	2	1	+	+
**Ch. *Eleocharito palustris*-*Sagittarion sagittifoliae***				
*Eleocharis ovata*	+	+	+	+
*Eleocharis palustris*	1	+	+	2
*Juncus articulatus*	2	1	+	1
*Rumex maritimus*	+		+	+
*Elatine hydropiper*	+	+		
*Limosella aquatica*	+	+		
**Ch. *Phragmito*-*Magnocaricetea***				
*Phragmites australis*	+	+	+	+
*Carex rostrata*	+	2	+	1
*Juncus effusus*	1	+	+	+
*Carex acuta*	+	+		+
*Lycopus europaeus*	+	+		1
*Glyceria fluitans*	+		+	+
*Cicuta virosa*	+	+		
*Sparganium erectum*	+			
**Accompanying species**				
*Agrostis stolonifera*	1	1	+	1
*Alopecurus geniculatus*	+	1	+	+
*Bidens tripartita*	+	+	+	+
*Juncus bulbosus*	+	1	1	+
*Mentha arvensis*	+		+	1
*Persicaria lapathifolia*	1		+	+
*Conyza canadensis*		+		+
*Gnaphalium uliginosum*	+	+		
*Juncus bufonius*	+	+		
*Potentilla norvegica*	+			+
*Ranunculus repens*			+	+
*Rorippa palustris*	1	+		
*Veronica scutellata*	+			+
*Carex hirta*				+
*Deschampsia cespitosa*				+
*Lythrum portula*		+		
*Persicaria minor*	+			
*Plantago major*				+
*Ranunculus reptans*	+			

## Hosting institution

Nature Research Centre, Institute of Botany, Žaliųjų Ežerų Str. 49, LT-12200 Vilnius, Lithuania
